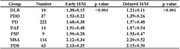# Iodine‐131‐meta‐iodobenzylguanidine (MIBG) cardiac scintigraphy in Neurodegenerative diseases

**DOI:** 10.1002/alz70857_101912

**Published:** 2025-12-25

**Authors:** Jie Sun, Qijun Li, Yanfeng Li, Ruixue Cui, Yong Ji

**Affiliations:** ^1^ Clinical College of Neurology, Neurosurgery and Neurorehabilitation, Tianjin Medical University, Tianjin, Tianjin, China; ^2^ Department of Nuclear Medicine, Peking Union Medical College Hospital, Beijing, Beijing, China; ^3^ Departmen of Neurology, peking union medical college hospital, Beijing, Beijing, China; ^4^ Department of Nuclear Medicine, Peking Union Medical College Hospital, Beijing, Beijing, China; ^5^ Department of Neurology, Tianjin Huanhu Hospital, Tianjin Key Laboratory of Cerebrovascular and neurodegenerative diseases, Tianjin dementia institute, Tianjin, Tianjin, China

## Abstract

**Background:**

Iodine‐131‐meta‐iodobenzylguanidine (^131^I‐MIBG) cardiac scintigraphy is a nuclear medicine technique used to assess the function of the sympathetic nervous system in the heart. Decreased myocardial ^131^I‐MIBG uptake has been reported in some patients with neurodegenerative diseases. The purpose of this study was to compare sympathetic denervation in the myocardium across various neurodegenerative diseases.

**Method:**

We recruited 222 patients with Parkinson's Disease (PD), 37 with Parkinson's Disease Dementia (PDD), 10 with Dementia with Lewy Bodies (DLB), 116 with Multiple System Atrophy (MSA), 14 with Pure Autonomic Failure (PAF), 9 with Progressive Supranuclear Palsy (PSP), and 63 with Parkinsonian Syndrome (PDS). The heart‐to‐mediastinum ratio (H/M ratio) at 15 minutes and 4 hours post‐injection of ^131^I‐MIBG cardiac scintigraphy was calculated and compared among the neurodegenerative disease groups mentioned above.

**Result:**

Patients with PD, PDD, and DLB had significantly lower H/M ratios for both early and delayed images than those with PAF, MSA, PSP, and PDS (*p* < 0.05). Moreover, patients with PDD and DLB demonstrated significantly reduced H/M ratios in both imaging sessions compared to those with PD (*p* < 0.05). However, no significant differences were observed between the PDD and DLB groups.

**Conclusion:**

^131^I‐MIBG cardiac scintigraphy is a valuable research tool for enhancing our understanding of autonomic nervous system involvement in neurodegenerative diseases. Patients with PDD and DLB showed lower cardiac MIBG uptake than those with PD. Our findings indicate a novel perspective on the potential differences in pathological changes among PD, PDD, and DLB.